# Practical tips for starting a successful national postgraduate course

**DOI:** 10.12688/mep.19636.4

**Published:** 2024-11-21

**Authors:** Magnus Sundbom

**Affiliations:** 1 Surgical Sciences, Uppsala Universitet, Uppsala, SE-75185, Sweden

**Keywords:** Postgraduate, Surgical education, Medicine, Learning, Upper abdominal surgery

## Abstract

**Background:**

Few start national courses, and those that do usually do it once. The aim of this paper is to outline an approach to conduct a successful national postgraduate course.

**Methods:**

The practical tips were derived from personal experience.

**Results:**

The 12 tips identified are: define learning needs and curriculum, create a functioning structure, recruit a committed faculty, obtain legitimacy, promote your course, try out the concept, establish administrative support, use modern techniques and accessories, create course-related social activities, keep all on board, collect ongoing evaluation, and stay in control.

**Conclusion:**

It is hoped that these tips will make it easier for others to take the decisive first step in the exciting task of starting a national course; that is: ‘to know the road ahead - ask those coming back’.

## Introduction

After completing more than seven years of undergraduate medical education and internship, a person applies for a five-year residency to become a specialist in general surgery in Sweden ^1^. During the national competence-controlled specialist training ^2^, the Swedish Surgical Society runs eight mandatory courses focusing on basic and organ-specific surgical training, all led by national faculties. Thereafter, when most surgeons choose to work within different subspecialties, no formal courses or national exams exist. This makes each hospital or surgical department responsible for the subsequent postgraduate education of its young specialists. This lack of organised professional training aroused my interest and prompted me to start a national postgraduate course for specialists in general surgery, specializing in upper abdominal surgery. To succeed, several difficulties and demands, for instance legitimacy, were to be overcome. Furthermore, since I lacked previous experience in starting and leading national courses, a short summary of solid tips would have been very valuable during the planning stage.

The following practical tips are intended to provide a framework when starting a postgraduate course of a higher level, based on the literature and the author’s own experience of running four such courses.

### Tip 1: Define learning needs and curriculum

For a new course it is essential to explore learner needs, ensuring the content attracts participants, as well as to attract high-quality teachers. Education expertise is very valuable, but these basic questions must be resolved first and can only be answered by yourself, having personal knowledge of the specific field ^3, 4^. Because a postgraduate course in upper abdominal surgery was lacking, both locally and nationally, the learning needs were unmet in my case. To set the learning objectives for a new course, one´s own professional competence and knowledge of key issues are important ^5^. Pedagogical expertise is invaluable in exploring different conceivable formats, but various structural issues such as existing skills and specific local learning needs must also be considered. Personally, I had to face the fact that forthcoming course participants worked either at a university hospital, doing advanced surgery in a specific field, or in a local hospital, doing more standardised high-volume procedures. Thus, the individual learning goals of the future participants must also be considered. To achieve this, the curriculum must be balanced to provide ‘S *omething for Everyone and Everything for Someone’.* To emphasise this, the statement was chosen as the motto for the first course.

An easy-to-use stepwise approach for curriculum development in medical education has been outlined ^6^. In short, the six steps describe problem identification and general needs assessment, needs assessment of targeted learners, goals and objectives, educational strategies, implementation, and evaluation and feedback. I found this focused approach very helpful, however, in retrospect I regret not using more educational support as this would have made my journey easier. A closer collaboration with an education expert would probably also have led to better course pedagogy. Instead, our curriculum was developed by me together with the faculty members, specialized in the various medical fields, during several meetings. We made sure that the course content was well above the requirements to become a specialist and asked the Swedish Surgical Association to review our curriculum proposal, as an external collaborative organisation. However, since we chose not to have a formal exam, thus lacking a defined outcome or exact level of knowledge, we could not apply constructive alignment. This approach to curriculum design, focused on closely aligning teaching and assessment to intended learning outcomes, is otherwise most important.

There is comprehensive research on the value of different educational delivery formats on knowledge retention ^7^, with face-to-face training, online learning, virtual classrooms, blended learning, and mobile learning being the five current trends ^8, 9^. In a recent systematic review, modern didactic formats such as flipped classroom, team-based learning, and ‘gamification’ resulted in superior engagement in millennial medical students compared to traditional teaching models ^10^. However, In our case, lecture-based education was chosen as a teaching principle because this concept is familiar to the target group of younger surgeons, educated before the start of problem-based learning ^11, 12^ and case studies ^13, 14^. Although we aimed to be student-focused, we did not let course participants with high knowledge in a specific topic be responsible for entire lectures, a peer-learning concept used with good results in other areas ^15^. Instead, we tried to increase the participants personal involvement in a specific topic by bringing clinical cases for discussion in class. In a recent systematic review, this type of team-based learning has been shown to enhance academic performance, communication skills, and clinical outcomes. It also strengthens learner engagement, motivation, and satisfaction compared to traditional lecture-based learning ^16^. Operative training, ruled out due to problems with time and medical liability, was replaced by videos and other How-I-do-it presentations. In a future revision, it would be interesting to include various simulations. A review from 2021 showed that a blended pedagogy including interactive technology-enhanced learning results in effective surgery training, by promoting knowledge sharing and skills learning ^17^. Finally, consider inviting one or two senior lecturers from other institutions or universities because this broadens the exchange of knowledge. Although staff-intensive, this set-up provides complementary depth and valuable external input to the discussed topic ^18^.

### Tip 2: Create a functioning structure

If the course structure is vague, it will not succeed, no matter how much work is put in. It is therefore important to consider not only what, but also who, when and where the education is provided. Firstly, decide whether the course should be digital or physical. Digital courses are more cost-effective ^19^ both for the organiser and the participants, because the meetings take place via a computer or other digital device. However, meeting participants and co-teachers creates the true course feeling with mutual exchange of thoughts and ideas, including outside of the classroom. Secondly, choose a suitable time and duration; a week-long course or multiple one-day meetings, perhaps on the same day of the week to maximise participation of all parties. We chose 15 one-day sessions, spread over three years, to lighten the course content. To ensure high attendance, avoiding posting course dates that conflict with other national meetings and courses. Thirdly, to ease travel planning during a longer course, particularly if it is held at different locations, identical schedules should be used every time. In our case, lectures started at 09:00 and to create a familiar atmosphere, refreshments were offered from 08:30, nearby the meeting room. Finally, consider dividing the teaching responsibility to reduce the workload and to obtain a larger knowledge base (three university hospitals in our case).

Lectures should be between 30–45 minutes, include a clear logical storyline with summaries or other cognitive breaks inserted every 15–20 minutes to renew attention ^20^. In the present postgraduate setting, we focused on evidence-based health care, implementing multifaceted and clinically integrated approaches with outcome assessment ^21, 22^. The chosen concept resulted in six lectures per day and allowed two short breaks and a one-hour lunch, common in our country. Allocated time for socializing is important as it encourages discussion and strengthens the bonds between the participants ^23^.

Determining the size of the course can be difficult as it concerns both financial and time considerations for each participant. As potential participants were used to seeing more applicants than available places, we limited the number of participants to 30, thus creating a good climate for interaction as well as a feeling of exclusivity. Furthermore, such an initiative probably attracts the correct type of participants, who will appreciate the new concept and give immediate, positive feedback.

### Tip 3: Recruit a committed faculty

Although the power of one person should never be underestimated, it is easier for a group of committed people to change the world. Thus, a highly competent faculty, with expert knowledge in each field, is needed to run a larger or diverse course. When recruiting, choose well-known individuals, i.e., national profiles; in my case I chose two from each of the three participating universities. The reputation of every participant was important to demonstrate the integrity of the course, facilitating its success. Furthermore, members of the faculty might be more prone to give engaging lectures than other staff. At the first faculty meeting, ensure that all are fully aware of the course structure and intention as well as the time involved. The input on the course content from these important individuals is crucial and results in a peer-review of the concept. The final course program with distribution of subjects and sites can be seen in Table 1. Moreover, remember that faculty members will require faculty development to keep them on board and ensure familiarity with later improvements and changes in the curriculum.

**Table 1.** Overview schedule for the new national postgraduate course in upper abdominal surgery, showing the 15 chosen subjects and the three university hospitals involved. All meetings take place on Fridays and the three May meetings, including the first gathering, are preceded by a social activity and dinner.

	Subject	University
**First year**		
September	Gastrointestinal bleedings	Stockholm
November	Acute pancreatitis	Linköping
January	Laparoscopy and gallbladder	Uppsala
March	Gastroesophageal cancer	Stockholm
May	Bariatric surgery	Linköping
**Second year**		
September	Hepatic cancer	Uppsala
November	Bile injuries and ERCP	Stockholm
January	Pancreatic cancer	Linköping
March	Advanced bariatric surgery	Uppsala
May	Liver metastases	Stockholm
**Third year**		
September	Benign gastroesophageal surgery	Linköping
November	Acute gastroesophageal conditions	Uppsala
January	Benign pancreatic disorders	Stockholm
March	Upper abdominal trauma	Linköping
May	Sarcomas and spleen	Uppsala

ERCP denotes Endoscopic Retrograde Cholangio Pancreatography

### Tip 4: Obtain legitimacy

To succeed to starting a national postgraduate course, it is vital to obtain legitimacy, in my case from the national surgical societies. Come well-prepared to such meetings, bringing the curriculum, a preliminary schedule for all meetings and your financial plan on printed pages. It is crucial to have these documents at hand while discussing various comments and questions after your presentation. If applicable, consider discussing other forms of support such as providing a web page ^24^, advertisement in their journals and future signing of course diplomas at the same meeting, as officials may change. The role of the national surgical societies was mainly formal accreditation, and by that quality assurance. In some cases, contact with higher accreditation bodies or official regulators may be needed ^25^. Be humble but not evasive because it is crucial for new courses to achieve accreditation; otherwise, they become unattractive to potential participants and their responsible managers. Remember that accreditation authorities share the same intent – to provide high-class teaching. An honest and early exchange of ideas should facilitate a good outcome.

### Tip 5: Promote your course

It is important to promote your course, as nobody else will. To start with, financiers of future participants should be informed about the aim and overall content of the course as well as the costs. The total cost will consist of the course fee and travel expenses but also the cost of missed production at the home hospital. Personal meetings are well-invested time because e-mails and mailed hardcopies easily disappear in the massive amount of information reaching decision makers. Support from both the profession and respective national societies are crucial for all tasks lying ahead and an important foundation. Online advertisements, in suitable channels, are important. Remember to post them well in advance of the upcoming start of the inaugural course and the surprisingly long time it takes for a submitted text to appear in a printed magazine. I boosted my first national course, by simultaneously publishing a short summary of the local try-out course ^26^. Finally, to promote your course in other contexts, add a short course description to all your appropriate professional presentations.

### Tip 6: Try out the concept

Despite meticulous planning, nothing is perfect until it has been tested. Before starting the first national course, I had an opportunity to run a limited local variant, testing the concept on a smaller scale ^26^. An authentic test is invaluable, not for checking the factual content, but for coordinating the content of presentations to avoid repetition and the overall schedule, i.e., balancing lectures, case presentations and breaks. The direct value of feedback from participants is also most important and resulted in several changes to the final program. Furthermore, these experiences convinced me that administrative support was crucial. As often test options in real life are scarce, input from colleagues and other teachers might have to be sufficient. However, remember that you can also test the layout and intellectual content of the lectures at various smaller meetings or during internal ongoing education. Yet, do not miss out on this important task as it is unpleasant to oversee a failing course, particularly your own.

### Tip 7: Establish administrative support

When starting and running a new course, there are multiple administrative tasks that must be performed. These range from registering applications and notifying course participants to the continuous need for sending material to all participants. If possible, get a disposed person to do this important work as the administration is more demanding than you initially envisage. Not only must upcoming meeting dates and schedules be distributed to the participants on time, but there is also a need to book meeting rooms, order lunch, and prepare the meeting room. Provide the finalized schedule on paper, paper and writing implements, and name tags, because this gives a serious impression. Place the course logo on the name badges as this unites the group and helps to promote the course in general. Well-functioning administrative support is also important for checking attendance, arranging faculty meetings, and much more. However, this adds administrative costs, costs not to be forgotten in the overall course budget. For your own good – do not skimp on this important task!

### Tip 8: Use modern techniques and accessories

Today virtually all communication, including distribution of articles and other information to students, is electronic. One possibility to handle this would be to create our own app. The large availability of platforms and other ready-made digital solutions have been addressed ^27^, and although this forum enables interactions between learners and educators, both groups need additional support to fully comprehend app functions ^28^. We chose a standard homepage to communicate with the students and faculty. After each meeting, remember to post a short summary of the content, including a reminder of the two forthcoming meetings ^24^. This leads to continuous contact with participants, while creating a nice showroom, not possible in an app-based solution.

However, most appreciated was the use of memory sticks, a cheap but very effective accessory. At the course start, all participants were given a personal memory stick, engraved with their name on one side, and the course name on the other side. As all presentations and cases were added onto the memory stick at the end of each meeting, the participants could pay attention to the presenter, instead of making notes on the spot. Later, they could easily check a detail, for example if being contradicted by a senior colleague at work. As a result of the continuous updating, everyone had a complete collection of all presented material at the course end. In a study Chahla *et al*., American residents reported they read more when provided with landmark scientific articles, fully sorted on a USB drive ^29^. For those who are technically interested, additional technologies such as various simulations using for example integrated simulators or virtual reality can be included ^30^. One could argue that our heavy use of traditional lectures contrasts with my appeal to use modern techniques, but unfortunately our course structure did not allow the use of additional technological devices.

### Tip 9: Create course-related social activities

Social interaction is important when acquiring new knowledge ^31^ and to increase interactions during class, it is important that participants feel comfortable and relaxed. Increased cohesion can easily be achieved by adding social activities such as a get-together dinner the night before the first meeting and by including free informal lunches at all meetings, your budget permitting. In addition, we decided to offer a social activity, e.g., visits to historical places and museums, roof-top walks and go-cart driving, the evening before the closing of each academic year. These optional activities were very popular and fulfilled additional course goals, namely creating an informal national network within the subspeciality as well as providing peer-to-peer learning opportunities.

### Tip 10: Keep all on board

During any long course, there is an increased risk of dropouts and general confusion. To counteract this, set the course dates well ahead, send reminders and deliver the detailed schedule for the next meeting at least one month in advance. If you have a diverse group, consider distributing selected state-of-the-art articles or other landmark literature to prepare participants for an upcoming meeting. By using this throughout the course, we ensured that all have a similar knowledge level and that teachers could engage with participants with a known predefined base. As a side effect, teachers were also forced to update their presentations according to the latest guidelines.

Another effective way to activate course participants between meetings is by letting some of them prepare a case of their own, for example condensed into a five-minute PowerPoint presentation. By regularly using this concept, we found the upcoming discussion with the rest of the group at the following meeting to increase the presenter´s interest in the subject as well as deepening his or her knowledge ^16^. In my course, this concept was highly appreciated by all. You can also use case presentations as an introductory part of a specific lecture; however, this requires that students submit their cases well in advance of the upcoming meeting. Furthermore, short quizzes ^32^ or web-based questions via smartphones ^33^ or physical clickers connected to a PowerPoint presentation ^34^ can be used to activate the participants during class, and, in our experience, this type of activity always led to good discussions.

If a long time passes between meetings for participants, it is even longer for the faculty. To keep faculty on board, welcome them to all meetings, and more importantly arrange biannual faculty meetings. At these meetings, present and discuss student evaluations in a constructive manner. This approach is crucial because faculty easily interpret evaluation feedback as a judgment, not just of their teaching ability, but of their personal and professional identity ^35^. For clinical teachers, several potential barriers can impede participation in teaching-improvement programs ^36^.

### Tip 11: Collect ongoing evaluation

**Formal** course evaluation is an integral part of the medical education enterprise ^37^. To oversee a course, especially a new one, student evaluations are crucial. If included from the onset, course participants perceive them as natural follow up after a meeting. Firstly, the responses will monitor the course and allow immediate improvements, from scheduling to all aspects of the content. If something does not work, do not try to justify it – make the necessary changes immediately! This should be done politely and in association with involved individuals. Secondly, and perhaps more important for a new course, the results can be used to demonstrate the value of the course to various stake holders and attract future participants. As the quality of responses received with web-based instruments is comparable to paper-based surveys ^38^, digital alternatives are preferable because of their simplicity. A course-specific app can be used, although such an app has not been shown better than web-based communication ^39^. At present, there are many suitable questionnaires online, which can be used free of charge ^40^. Take care to make your survey short and straightforward ^38^, for example by using Likert scales, and let an independent person complete the first questionnaire before distributing it to all participants. At the next meeting, remember to thank participants for the survey responses and take the opportunity to comment on significant observations, without revealing the results in detail. In this course I have not worked with teacher evaluations but have good experience of this in undergraduate education ^41^.

The present course evaluations have been positive, with more than 80 % of the participants rating the individual meetings as being four or better on a five-degree Likert scale (five best). In our case, this formative assessment was mainly used to identify areas of improvement, such as optimised time slots for specific lectures, exchanged teachers, and new topics, the latter with increased focus on practical training. **As a result of the continuous work with the course content right from the start, we have had a waiting list for the last six years. This, in combination with my education award from the Swedish Surgical Society indicates that the course has become a credible national program**.

### Tip 12: Stay in control

As course leader, it is essential to be in control, particularly during the introduction phase of a new course. Be present at all meetings, welcome the participants and take the lead. Intervene politely if the program is running behind schedule or gets stuck on a topic. To keep everything in good working order, scrutinise the upcoming program and other course arrangements before they are sent out to participants. Your course and you are constantly being judged by the accuracy and timing of such documents. As mentioned above, regular faculty meetings, for example held during ordinary course days to minimize travel, will lead to improvements in the curriculum as well as increased cohesion in the teacher group.

Furthermore, it is important to live up to the set requirements. If you set a minimum attendance (75% in our case) for an approved course, you must have a system for recording attendance at every meeting. This is particularly important in courses lasting several years due to the delay making up for missed meetings will be long. As a course leader, it is important that you quickly answer all types of questions from course participants as well as other interested parties. Your behaviour will be associated with the reputation of the course. Finally, remember to communicate your successes outwards, for example in job-related journals ^26^ and at professional meetings, as nothing could be more wrong than the old saying ‘No news is good news’.

## Conclusion

It is rewarding to start a new educational undertaking; however, the road ahead can be tricky. I hope that my tips can make it easier for others to take the decisive first step; as someone said: ‘to know the road ahead - ask those coming back’.

In short, develop your specific course program, aim high and engage early with stakeholders and accreditation authorities. Listen respectfully to all points of view and learn from the critics – but stick to your intention. **Collaborate with an education expert to get optimal course pedagogy**. Include a straightforward course evaluation from the beginning and disseminate the results as soon as possible. Keep all on board by maintaining the control yourself.

## Data Availability

No data are associated with this article.
