# Supplementary material for: Practical tips for starting a successful national postgraduate course
Source: MedEdPublish (2016). 2024 Nov 21;13:26. Originally published 2023 May 12. [Version 4] doi: 10.12688/mep.19636.4 (PMC11733731; doi:10.12688/mep.19636.4)
Supplement: Peer review history

# Reviewer report: Reviewer response for version 4

**DOI:** 10.21956/mep.22035.r40766

**Published:** 2025-01-13

**Authors:** Lisa Graves

**Affiliations:** 1 Western Michigan University, Kalamazoo, Michigan, USA

This is a well constructed personal report about curriculum development.  

 Introduction is well constructed.  It would be helpful for the reader to have clarity about the structure of training in Sweden. Is there a subspecialty in upper abdominal surgery?  

- Tip 1: This tip describes a team developing the curriculum.  It would be helpful to have this team described.
- Tip 3: It might be useful to indemnify the actual number of faculty for the entire course. Did each develop their own material/did they use shared objectives.
- Tip 4: Consider describing the accreditation process for the curriculum.
- Tip 6: A description of the evaluation of both the course and the pilot would be useful to readers.
- Tip 8: Readers may ask about the use of USB sticks rather than using an online learning management system.
- Tip 10: Consider clarity around “my course” versus “the course”.
- Tip 11: Consider sharing the evaluation tool and indicating if the evaluations have included multiple levels of evaluation.
- Tip 12: Staying in control is accurate but consider changing this to something that reflects the importance of presence.

If evidence from practice is presented, are all the underlying source data available to ensure full reproducibility?

Yes

Is the topic of the practical tips discussed accurately in the context of the current literature

Yes

Are all factual statements correct and adequately supported by citations?

Yes

Are the conclusions drawn balanced and justified on the basis of the presented arguments?

Yes

Are arguments sufficiently supported by evidence from the published literature and/or the authors’ practice?

Yes

Reviewer Expertise:

UGME/curriculum

I confirm that I have read this submission and believe that I have an appropriate level of expertise to confirm that it is of an acceptable scientific standard, however I have significant reservations, as outlined above.

# Reviewer report: Reviewer response for version 4

**DOI:** 10.21956/mep.22035.r40697

**Published:** 2025-01-07

**Authors:** Claire Stocker

**Affiliations:** 1 Aston Medical School, Aston University, Birmingham, England, UK

The manuscript is an interesting paper presenting twelve recommendations for establishing a national postgraduate surgical course, drawing from the author's experience. Revisions have incorporated earlier feedback, such as the emphasis on educational expertise and participant feedback, but several issues remain unaddressed. The manuscript's reliance on lectures as the primary teaching method lacks justification. Although there is a brief acknowledgement of more interactive, learner-centered approaches, such as team-based learning, the rationale for favouring lectures remains underdeveloped. Justification for these pedagogical choices, supported by evidence or comparative analyses, would enhance the manuscript.

 The section on course evaluation also requires improvement. Despite Tip 11 underscoring the importance of feedback, the manuscript lacks data or examples demonstrating the course's effectiveness. Integrating quantitative or qualitative evaluation results would substantiate the recommendations and reinforce their credibility. The acknowledgement of the absence of formal assessment methods is not compensated for by alternative evaluative approaches.

 The manuscript's engagement with broader educational theories, such as constructive alignment and co-design, is superficial. Incorporating advancements in educational innovation, including technology-enhanced learning and interactive methodologies, would elevate the manuscript's relevance and contribution. Although the manuscript's language is clear and professional, certain sections exhibit repetitiveness, particularly in justifying the utilisation of traditional lectures. The conclusion could discuss the broader applicability of the recommendations to other specialities and international contexts.

If evidence from practice is presented, are all the underlying source data available to ensure full reproducibility?

Not applicable

Is the topic of the practical tips discussed accurately in the context of the current literature

Yes

Are all factual statements correct and adequately supported by citations?

Yes

Are the conclusions drawn balanced and justified on the basis of the presented arguments?

Partly

Are arguments sufficiently supported by evidence from the published literature and/or the authors’ practice?

Yes

Reviewer Expertise:

Professionalism, Curriculum design

I confirm that I have read this submission and believe that I have an appropriate level of expertise to confirm that it is of an acceptable scientific standard, however I have significant reservations, as outlined above.

# Reviewer report: Reviewer response for version 4

**DOI:** 10.21956/mep.22035.r40229

**Published:** 2024-11-25

**Authors:** John Cookson

**Affiliations:** 1 University of Portsmouth, Portsmouth, England, UK

I think the previous comments and concerns have now been addressed.

If evidence from practice is presented, are all the underlying source data available to ensure full reproducibility?

Partly

Is the topic of the practical tips discussed accurately in the context of the current literature

Partly

Are all factual statements correct and adequately supported by citations?

Partly

Are the conclusions drawn balanced and justified on the basis of the presented arguments?

Partly

Are arguments sufficiently supported by evidence from the published literature and/or the authors’ practice?

Partly

Reviewer Expertise:

medical education particularly assessment

I confirm that I have read this submission and believe that I have an appropriate level of expertise to confirm that it is of an acceptable scientific standard.

# Reviewer report: Reviewer response for version 3

**DOI:** 10.21956/mep.21703.r36147

**Published:** 2024-06-12

**Authors:** John Cookson

**Affiliations:** 1 University of Portsmouth, Portsmouth, England, UK

It would like to have had a bit more detail on what, if anything, the author has missed by not engaging with a pedagogical expert.

If evidence from practice is presented, are all the underlying source data available to ensure full reproducibility?

Partly

Is the topic of the practical tips discussed accurately in the context of the current literature

Partly

Are all factual statements correct and adequately supported by citations?

Partly

Are the conclusions drawn balanced and justified on the basis of the presented arguments?

Partly

Are arguments sufficiently supported by evidence from the published literature and/or the authors’ practice?

Partly

Reviewer Expertise:

medical education particularly assessment

I confirm that I have read this submission and believe that I have an appropriate level of expertise to confirm that it is of an acceptable scientific standard, however I have significant reservations, as outlined above.

# Response

**Published:** 2024-08-02

**Authors:** Magnus Sundbom

**Affiliations:** Surgical Sciences, Uppsala Universitet, Uppsala, Uppsala County, Sweden

Thank you for your comments, I concur. In the updated version, the importance of educational support has been highlighted. In Tip 1, I have added the large value of education expertise, and stated that I regret not using more educational support during the setup phase, as this both would have made my journey easier and resulted in better course pedagogy.

# Reviewer report: Reviewer response for version 3

**DOI:** 10.21956/mep.21703.r36148

**Published:** 2024-04-08

**Authors:** Richard B. Hays

**Affiliations:** 1 College of Medicine and Dentistry, James Cook University, Townsville, Qld, Australia

Although I accept that anyone can develop a training program and that trainees will attend (often more to fear of missing out than other factors) I do not agree that education expertise is not required to do this.  As an educator I would be asking questions about community need, learner needs, evolution of practice (diagnostic and management) and exploring a variety of formats.  Formal evaluation is also important.  The amendments have not really addressed these points.  I think the challenge is how to convert the work so far into a credible, high quality, effective national training program.

If evidence from practice is presented, are all the underlying source data available to ensure full reproducibility?

Yes

Is the topic of the practical tips discussed accurately in the context of the current literature

Partly

Are all factual statements correct and adequately supported by citations?

Yes

Are the conclusions drawn balanced and justified on the basis of the presented arguments?

Partly

Are arguments sufficiently supported by evidence from the published literature and/or the authors’ practice?

Partly

Reviewer Expertise:

Medical education

I confirm that I have read this submission and believe that I have an appropriate level of expertise to state that I do not consider it to be of an acceptable scientific standard, for reasons outlined above.

# Response

**Published:** 2024-08-02

**Authors:** Magnus Sundbom

**Affiliations:** Surgical Sciences, Uppsala Universitet, Uppsala, Uppsala County, Sweden

Thank you for your continuous work and patience with my manuscript. In the updated version, the importance of educational support has been highlighted.

 In Tip 1, I have added the large value of education expertise, and stated that I regret not using more educational support during the setup phase, as this both would have made my journey easier and resulted in better course pedagogy. I have also echoed your thoughts on asking questions about learner needs and exploring various formats.

 In tip 11, I have emphasized the need of formal evaluation and described why I believe that my course has turned into a credible national program. Considering the long review process, starting in March 2023, I sincerely hope that my paper can finally be accepted for publication.

# Reviewer report: Reviewer response for version 2

**DOI:** 10.21956/mep.21481.r35935

**Published:** 2024-02-23

**Authors:** John Cookson

**Affiliations:** 1 University of Portsmouth, Portsmouth, England, UK

This is an account of a new postgraduate course in specialist upper abdominal surgery set up in Sweden to meet a perceived need in that country. It is framed as 12 tips for non-pedagogical experts.

 Tip 1 This section could be strengthened by a consideration of the concept of constructive alignment. Thus the learning process that is selected depends on the outcomes that have been set. The assessment then measures whether those has been achieved. The term ‘outcomes’ is not mentioned although ‘objectives’ is.  It seems probable that the objectives were developed in house. It would be helpful to know if other national or international outcomes/objectives were consulted. Assessment is not mentioned in this section.  Technology-enhanced learning is mentioned but, oddly, not simulation. 

 Tip 2 Despite Tip 1 identifying several good learning strategies, this section is mainly concerned with lectures. There are 6/day which implies that they take most of the time. Small group learning is noted in Tip 1 but not here, although small group learning is more complex to organise than lectures.

 Tip 3 It would be helpful to know how far the faculty were involved in constructing the design and objectives of the course so as to encourage ‘buy in’.

 Tip 4 The role of the national surgical societies is not clear.  Are they providing comment, direct input, formal accreditation or quality assurance?

 Tip 5 The importance of direct contact rather than email or hard copy is well made. Costs are mentioned here but could perhaps have a fuller treatment.

 Tip 6 Does not mention the value of feedback from participants although evaluation is in 11

 Tip 7 Is an opportunity to consider administrative costs

 Tip 8 An appeal to use modern techniques contrasts with the heavy use of traditional lectures

 Tip 9 Could point out that social activities are also learning opportunities

 Tip 10 This brings in several other pedagogical activities but it is not clear how much they were used and how they were integrated into the rest of the course

 Tip 11 On evaluation makes several important points. Formative assessment is not mentioned

 Tip 12 The importance of visible leadership is well made

If evidence from practice is presented, are all the underlying source data available to ensure full reproducibility?

Partly

Is the topic of the practical tips discussed accurately in the context of the current literature

Partly

Are all factual statements correct and adequately supported by citations?

Partly

Are the conclusions drawn balanced and justified on the basis of the presented arguments?

Partly

Are arguments sufficiently supported by evidence from the published literature and/or the authors’ practice?

Partly

Reviewer Expertise:

medical education particularly assessment

I confirm that I have read this submission and believe that I have an appropriate level of expertise to confirm that it is of an acceptable scientific standard, however I have significant reservations, as outlined above.

# Response

**Published:** 2024-03-02

**Authors:** Magnus Sundbom

**Affiliations:** Surgical Sciences, Uppsala Universitet, Uppsala, Uppsala County, Sweden

Thank you for your valuable comments and constructive suggestions.

 Tip 1. Our problem with applying constructive alignment has been added as well as the lack of formal outcome. The objectives were developed in house by me and the faculty, whereafter the course content was examined and approved by our national societies. The possible use of simulation has been added.

 Tip 2. I agree that despite identifying several good learning strategies, we still ended up with traditional lectures, taking most of the time. The underlying reason for this (well-known concept to the course participants and easy to organize) has already been stated in the text. Tip 3. The importance of involving the faculty in constructing the design and objectives of the course has been added.

 Tip 4. The role of the national surgical societies was mainly formal accreditation, and by that quality assurance. This information has been added.

 Tip 5. I have elaborated more on costs, also including the cost of missed production at the home hospital.

 Tip 6. The large importance of feedback from participants has been added.

 Tip 7. Administrative costs are now more thoroughly discussed, adding the importance to included them in the overall budget.

 Tip 8. The contrast between my appeal to use modern techniques and the present heavy use of traditional lectures has been commented.

 Tip 9. Thank you, the fact that social activities are learning opportunities has been added.

 Tip 10. The present use of the mentioned pedagogical activities has been added as well as the fact that they were used throughout the course.

 Tip 11. Our problem with using formative assessment has been added.

 Tip 12. Thank you for finding the stated importance of visible leadership well made.

# Reviewer report: Reviewer response for version 2

**DOI:** 10.21956/mep.21481.r35468

**Published:** 2023-12-21

**Authors:** Richard B. Hays

**Affiliations:** 1 College of Medicine and Dentistry, James Cook University, Townsville, Qld, Australia

Thank you for responding and revising the manuscript.  I am still not sure that it reaches the standard required - that of an innovation that has been shown to fill an identified gap, be useful and effective (better outcomes).  It does report an interesting, 'ground up' reaction to an identified need, but the presentation as a personal journey without clear evidence of curriculum development and evaluation makes this more of a newsletter article. As the program continues it should be possible to gather sufficient data for evaluation - at that stage this could potentially be a much more valuable contribution.

If evidence from practice is presented, are all the underlying source data available to ensure full reproducibility?

Yes

Is the topic of the practical tips discussed accurately in the context of the current literature

Partly

Are all factual statements correct and adequately supported by citations?

Yes

Are the conclusions drawn balanced and justified on the basis of the presented arguments?

Partly

Are arguments sufficiently supported by evidence from the published literature and/or the authors’ practice?

Partly

Reviewer Expertise:

Medical education

I confirm that I have read this submission and believe that I have an appropriate level of expertise to confirm that it is of an acceptable scientific standard, however I have significant reservations, as outlined above.

# Response

**Published:** 2024-03-02

**Authors:** Magnus Sundbom

**Affiliations:** Surgical Sciences, Uppsala Universitet, Uppsala, Uppsala County, Sweden

Thank you! My purpose in this article is to show that it is possible for non-pedagogical experts to start a new course and to help them with practical tips, based on my own experiences as I groped my way. I understand your doubts concerning the lack of effectiveness, e.g., improved outcomes on a larger scale, a result that we never hoped to achieve by launching the present course. Of course, we strived for increased knowledge of our specialists, but also to create an increased national cooperation and solidarity between participants from different hospitals. By time, the course and its content has improved, as demonstrated by feedback from the participants. However, I do not believe that a detailed presentation of these data would be of interest for the reader. To my great delight, the article has already been downloaded and read by several people.

# Reviewer report: Reviewer response for version 1

**DOI:** 10.21956/mep.21035.r35261

**Published:** 2023-11-24

**Authors:** Richard B. Hays

**Affiliations:** 1 College of Medicine and Dentistry, James Cook University, Townsville, Qld, Australia

This article documents the journey of a small team led by one individual in making a significant change in postgraduate surgical specialty education in Sweden. This may be both a strength and a weakness. Other specialty programs in Sweden and other countries with a similar training system may find this helpful, but I am not sure that it will be of much value elsewhere.  The usual system is that there is mandatory training and experience, with a national curriculum and national assessment on top of WBA – a world away from the context of this paper. 

 The list of monthly topics is small. I am concerned that the ‘curriculum’ was based on one individual’s professional experience, rather than a carefully thought through process involving employers, learners and teachers and using prevalence and outcomes data.  More information on how the curriculum was developed could be provided. 

 Another concern is the format, which seems reliant on lectures with some case presentations. 

 Still another is the ‘stay in control’ tip, which risks being seen as paternalistic.  It may be better for the learners (trainees) to have more control over delivery, in keeping with the current ‘co-design’ trend and sharing responsibility for learning. 

 The project will need careful evaluation – are there different outcomes for those who participate vs those who do not?  If evidence for this emerges should the program be made mandatory, not just locally but also nationally?  I suspect that proving that may be difficult in such an open system.  

 I am not concerned about the course being run – education is always a good idea – but not convinced that it reflects best practice across curriculum design, content and delivery.

If evidence from practice is presented, are all the underlying source data available to ensure full reproducibility?

Yes

Is the topic of the practical tips discussed accurately in the context of the current literature

Partly

Are all factual statements correct and adequately supported by citations?

Yes

Are the conclusions drawn balanced and justified on the basis of the presented arguments?

Partly

Are arguments sufficiently supported by evidence from the published literature and/or the authors’ practice?

Partly

Reviewer Expertise:

Medical education

I confirm that I have read this submission and believe that I have an appropriate level of expertise to confirm that it is of an acceptable scientific standard, however I have significant reservations, as outlined above.

# Response

**Published:** 2023-11-28

**Authors:** Magnus Sundbom

**Affiliations:** Surgical Sciences, Uppsala Universitet, Uppsala, Uppsala County, Sweden

Thank you for your valuable comments. Please see my point-to-point response below. The stated changes have been included in the revised version of the manuscript.

 Comments on paragraph #1. The described course is a non-mandatory course for specialists with an extra interest in the field, thus completly outside the official specialty program. Despite this, all course rounds have been full and we have an increasing waiting list.

 Comments on paragraph #2. The curriculum was not based on one individual’s professional experience, but developed by the faculty. e.g., seven specialists in the field, and then reviewed by the Swedish Surgical Association, as an external collaborative organisation. This has been explained in the revised manuscript.

 Comments on paragraph #3. I agree, however, as the the ‘stay in control’ tip is aimed at future course leaders, not participants, I really wanted to stess this very important point. Without having full control - no lasting course.   

 Comments on paragraph #4. Comparing the outcomes for those who participate vs those who do not is an intriguing idea, however, I believe that it will be difficult to find suitable outcome varaiables. The high level of interest in the course (>100 participants so far) and the attention of my educational prize in 2021 has contributed to a sense that the course is seen as almost mandatory by heads of surgical departments.

# Reviewer report: Reviewer response for version 1

**DOI:** 10.21956/mep.21035.r33342

**Published:** 2023-05-25

**Authors:** Sophie Lumley

**Affiliations:** 1 NHS England, Redditch, England, UK

Thank you for submitting this ‘Practical Tips’ article. It details 12 tips for running a national course, based on the author’s experience of running 4 national courses, aimed at those subspecialising within general surgery, in Sweden. The author states the benefits and rewarding nature of running these courses, but provides a checklist of tips to consider when navigating the legitimacy, content and delivery of a new course.

 My recommendation is to approve this article with some reservations. I have the following feedback.

- I can see a clear justification for the development of these courses, and commend the author in his success in this. However, it would be useful to have a clear justification for this article. What gap in the literature is this this article filling. What information and advice are already available to support the set up and delivery of national courses? What is this article adding? What resources did the author draw on in early stages of the process of setting up this course.
- I felt at times, particularly in Tips 2 and 3, some statements of fact weren’t clearly evidenced with citation or justification from experience. E.g., “ *There is little evidence that different curricula lead to different outcomes, curriculum development should integrate a content area with educational theory and methodology,*” “ *Digital courses are less demanding, both for the organiser and the participants*” and “ *members of the faculty are more prone to give engaging lectures than other staff*”
- I found most arguments were reasonably well justified, however some were slightly weaker – E.g. “ *In our case, lecture-based education was chosen as a teaching principle because this concept is familiar to the target group of younger surgeons, educated before the start of problem-based learning and case studies*” –There is comprehensive research on the value of different educational delivery formats, and I didn’t feel the use of lectures was clearly justified, and for those seeking to develop their own national programmes they may have other considerations which may be useful to highlight here. I agree reference 6, cited with this statement, is a helpful reference regarding curriculum development, and perhaps could be expanded on and how it was applied in this context, and could be applied in others.
- Whilst there was mention of the importance of evaluation of a national course, no clear results from evaluations were included in this article to demonstrate the impact of these courses. This also may have helped provide evidence to justify the structure, delivery and content of these courses. Linked to this it would be useful to understand the future plan for these courses, as a marker of success of the initial stages.

If evidence from practice is presented, are all the underlying source data available to ensure full reproducibility?

Partly

Is the topic of the practical tips discussed accurately in the context of the current literature

Partly

Are all factual statements correct and adequately supported by citations?

Partly

Are the conclusions drawn balanced and justified on the basis of the presented arguments?

Partly

Are arguments sufficiently supported by evidence from the published literature and/or the authors’ practice?

Partly

Reviewer Expertise:

I'm a General Practitioner in the UK. I'm currently a Training Programme Director for GP training across Shropshire for 130+ GP trainees in the region. I have previously published research on: links between extracurricular activities and academic success at medical school; the role of virtual reality as a tool in medical education and a quality improvement project to overall regional GP training with the use of peer facilitation and case based discussion. I have a PGCert in Clinical Education and FHEA status.

I confirm that I have read this submission and believe that I have an appropriate level of expertise to confirm that it is of an acceptable scientific standard, however I have significant reservations, as outlined above.

# Response

**Published:** 2023-11-28

**Authors:** Magnus Sundbom

**Affiliations:** Surgical Sciences, Uppsala Universitet, Uppsala, Uppsala County, Sweden

Thank you for your valuable comments. Please see my point-by-point response below. The text has been updated accordingly.

 Comments in paragraph #1: To justify the article, I have added the sentence 'Furthermore, since I lacked previous experience in starting and leading national courses, a short summary of solid tips would have been very valuable during the planning stage.'

 Comments in paragraph #2: The not clearly evidenced statements have been removed ('Little evidence that different curricula lead to different outcomes') or rephrased ('Digtal courses' and 'Mebmers of the faculty').

 Comments in paragraph #3: The paragraph on curriculum development has been rewritten. It now includes the fact that there is comprehensive research on the value of different educational delivery formats on knowledge retention, and the forms are exemplified by a modern version. Four new references have been added. The contence of reference 6 has been expanded and mirrored in the context.

 Comments in paragraph #4: Results from our course evaluations have been presented, however, briefly as I do not concider them to be a main part of the content of this paper.
